# Immuno-Virological Discordance and the Risk of Non-AIDS and AIDS Events in a Large Observational Cohort of HIV-Patients in Europe

**DOI:** 10.1371/journal.pone.0087160

**Published:** 2014-01-31

**Authors:** Alexander Zoufaly, Alessandro Cozzi-Lepri, Joanne Reekie, Ole Kirk, Jens Lundgren, Peter Reiss, Djordje Jevtovic, Ladislav Machala, Robert Zangerle, Amanda Mocroft, Jan Van Lunzen

**Affiliations:** 1 Department of Medicine I, Infectious Diseases Unit, University Medical Center Hamburg-Eppendorf, Hamburg, Germany; 2 Department of Infection and Population Health, University College London, London, United Kingdom; 3 Copenhagen HIV Programme - Department of Infectious Diseases and Rheumatology, section 8632, Rigshospitalet, University of Copenhagen, Copenhagen, Denmark; 4 University of Amsterdam, Academic Medical Center, Department of Global Health, and Stichting HIV Monitoring, Amsterdam, Netherlands; 5 University of Belgrade School of Medicine Infectious Diseases Hospital, HIV/AIDS Department, Belgrade, Serbia; 6 Department of Infectious Diseases, Third Faculty of Medicine, Charles University Prague, Prague, Czech Republic; 7 Medical University of Innsbruck, Department of Dermatology and Venereal Diseases, Innsbruck, Austria; University of Athens, Medical School, Greece

## Abstract

**Background:**

The impact of immunosuppression despite virological suppression (immuno-virological discordance, ID) on the risk of developing fatal and non-fatal AIDS/non-AIDS events is unclear and remains to be elucidated.

**Methods:**

Patients in EuroSIDA starting at least 1 new antiretroviral drug with CD4<350 cells/µl and viral load (VL)>500 copies/mL were followed-up from the first day of VL< = 50 copies/ml until a new fatal/non-fatal non-AIDS/AIDS event. Considered non-AIDS events included non-AIDS malignancies, pancreatitis, severe liver disease with hepatic encephalopathy (>grade 3), cardio- and cerebrovascular events, and end-stage renal disease. Patients were classified over time according to whether current CD4 count was above (non-ID) or below (ID) baseline level. Relative rates (RR) of events were calculated for ID vs. non-ID using adjusted Poisson regression models.

**Results:**

2,913 patients contributed 11,491 person-years for the analysis of non-AIDS. 241 pre-specified non-AIDS events (including 84 deaths) and 89 AIDS events (including 10 deaths) occurred. The RR of developing pre-specified non-AIDS events for ID vs. non-ID was 1.96 (95% CI 1.37–2.81, p<0.001) in unadjusted analysis and 1.43 (0.94–2.17, p = 0.095) after controlling for current CD4 count. ID was not associated with the risk of AIDS events (aRR 0.76, 95% CI 0.41–1.38, p = 0.361).

**Conclusion:**

Compared to CD4 responders, patients with immuno-virological discordance may be at increased risk of developing non-AIDS events. Further studies are warranted to establish whether in patients with ID, strategies to directly modify CD4 count response may be needed besides the use of ART.

## Introduction

Combination antiretroviral therapy (cART) mainly exerts its effect on the reduction of HIV related morbidity and mortality by suppressing HIV viral load which leads to an increase of CD4 cells [1], [2]. A rapid increase of CD4 cells is important in the light of the fact that a significant proportion of patients continue to present for therapy when advanced immunodeficiency is present and their immediate risk of AIDS and death is high [3]. The majority of patients achieve a sustained reconstitution of CD4 cells upon starting cART [4] although blunted CD4 responses do also occur in patients initiating when their CD4 count was <200 cells/µl [5], [6], [7]. The inability to achieve an increase in CD4 count in the context of sustained virological suppression is also known as immuno-virological discordance (ID) [8] and has been associated with increased mortality [9]. Indeed, previous work from the ClinSurv Study group showed an association between ID and the risk of AIDS and death [10]. However, this risk was greatly reduced with longer time spent with a suppressed viral load suggesting that maintaining a suppressed viral load is key for patients with immuno-virological discordance [10]. Previous data indicate that non-AIDS events are nowadays important contributors to HIV associated morbidity and mortality and that current CD4 is also a predictor of these events [11]. The role of immuno-virological discordance to predict the risk of fatal and non-fatal non-AIDS and AIDS events remains unclear and was evaluated in this analysis. The aim of our analysis was to investigate whether a lack of any increase in CD4 count above levels observed prior to starting a suppressive regimen and despite evidence of sustained viral suppression was associated with adverse clinical progression.

## Methods

### EuroSIDA Cohort

The EuroSIDA study is a prospective, observational cohort study including HIV-1 infected patients from 31 European countries as well as Israel and Argentina [12].

To date, nine cohorts have been enrolled starting in May 1994. At enrollment, demographic characteristics and clinical information are obtained. Every 6 months CD4 count and viral load data as well as data on antiretroviral drugs used are collected in a standardized report form and forwarded to the coordinating center. The pre-specified non-AIDS events included are those used in previous EuroSIDA publications on this topic [11] and the expert opinion of members of the steering committee as events which are likely to have a major impact of HIV associated morbidity and mortality. These data have been routinely collected since January 2001 and include non-AIDS malignancies, pancreatitis, severe liver disease with hepatic encephalopathy (grade 3 or 4), cardio- and cerebrovascular events (i.e. myocardial infarction, placement of coronary artery bypass graft or angioplasty, carotid endarterectomy, stroke), and end-stage renal disease. Death from all causes except for AIDS related conditions was counted as non-AIDS defining. Diagnoses of AIDS are recorded using the clinical 1993 CDC definition [13] and causes of deaths are categorized using the Coding Cause of Death in HIV (CoDe) classification [14]. Members of the coordinating office visit all centers at least once a year to ensure correct patient selection and to verify that data are accurate. In patients with a clinical event, all entered data are checked against case files and 10% of case files are checked in all other patients.

### Ethics Statement

Each of the centers participating in EuroSIDA sought ethical clearance according to local regulations and laws and patients gave their informed consent to take part in the study. All data were anonymized prior to statistical analysis. The senior investigator at each clinical site is responsible for obtaining and maintaining this/these approval(s) at all times during the conduct of the study. Full study details are available at www.cphiv.dk.

### Patient Selection

Patients in the cohort with a CD4 count of less than 350 cells/µl who after 1 January 2001 started at least one new antiretroviral drug with most recent viral load >500 copies/mL (either because starting their first cART or because switching drugs after failing on their previous regimen) and subsequently achieved viral suppression < = 50 copies/mL were included. Follow-up accrued from the first day of viral suppression below 50 copies/mL following the initiation of the new regimen (baseline) until the first of either a fatal or non-fatal non-AIDS or AIDS event, viral rebound >50 copies/mL (defined as the time of the first of 2 consecutive values above this threshold) or patients’ latest available follow-up visit in which they were known to be still event-free. Each AIDS and non-AIDS condition which developed during follow-up was counted as one event and recurrences of the same condition were not counted.

Data were left-censored at entry in the cohort and patients had to have at least one month of prospective follow-up after baseline to be included. CD4 counts were carried forward until a new CD4 test was performed.

### Statistical Methods

Immuno-virological response was defined using both the CD4 count measured prior to initiation of the suppressive regimen and the current CD4 count. In particular if patients’ current CD4 count was still equal or below the pre-ART initiation level then the person was defined as immuno-virological discordant (ID) at the time of the current CD4 count measure. In contrast, any current rise in CD4 count above pre-ART level would reverse his/her classification to immunological responder (non-ID). Rates of fatal and non-fatal non-AIDS and AIDS events were calculated over 6 monthly periods (0–6, 7–12, 13–18, 19–24, >24 months) of virological suppression after baseline and stratified according to ID status. Univariable and multivariable Poisson regression models were performed to estimate the risk associated with a current ID status (fitted as a time-dependent covariate). When exploring the magnitude of the risk associated with being ID over time, a categorical covariate grouping periods of follow-up with thresholds 0–6, 7–12, 13–18, 19–24, >24 months and using the 0–6 months period as the comparator group was included in the model. Other potential risk factors or confounders which were considered for inclusion in the model were: age, hepatitis C serostatus, mode of HIV transmission, race, cohort of origin, the number of previous ART regimens used, current CD4 count (or baseline CD4 count in an alternative model) and specific antiretroviral drugs included in the newly initiated regimen.

Potential confounders were selected on the basis of prior knowledge and when hypothesized to be common causes of both the exposure of interest and the risk of developing the outcome. A software based on directed acyclic graphs [15] was employed to identify the appropriate model to correctly estimate the direct effect of ID condition on the risk of the outcomes.

To be consistent with previous analysis of the data of the cohort, current CD4 count was fitted on a log2 scale as this was shown to improve the goodness of fit of the model. Models also included time from initiation of new regimen to viral suppression, current or previous smoking, history of impaired renal function at baseline (defined as an estimated Cockroft-Gault glomerular filtration rate <60 mg/dL/1.73 m^2^), anaemia (defined as haemoglobin levels <14 mg/dl in males and <12 mg/dl in females), diabetes (defined as having previously started any antidiabetic treatment or diagnosis of insulin dependent diabetes), and hypertension (defined as a systolic/diastolic blood pressure> = 140/> = 90 mmHg mmHg or previous initiation of any antihypertensive treatment).

Separate analyses were performed for two endpoints (fatal and non-fatal non-AIDS, and fatal and non-fatal AIDS) using a competing risk approach (i.e. by truncating follow-up at July 01, 2011 instead of the date at which a competing event occurred). In brief, the competing event (e.g. a non-AIDS event in the case of a cause specific analysis with an AIDS event as endpoint) was removed to avoid overestimation of the cumulative probability of AIDS events [16]. The cause of death was used to define the non-AIDS (only deaths from any cause that were unrelated to AIDS are included) and AIDS (only deaths from AIDS related causes are included) endpoints. We assumed that deaths due to other reasons (e.g. suicide) did not have an indirect effect on the probability to develop AIDS or other non-AIDS co-morbidities and therefore patients’ follow-up was censored at the date of death for these reasons. Of note, because we adopted combined endpoint such as ‘AIDS events’ the issue of competing risk of developing, say, a PCP prior to a diagnosis of, say, Kaposi sarcoma is automatically removed. For the non-AIDS endpoint we performed sensitivity analyses in the subsets of patients who were treatment-naïve when they started the new baseline regimen and of those who achieved complete viral suppression within a maximum of 1 year after starting the new baseline regimen. The latter analysis aimed at reducing potential bias introduced by participants who may have experienced adherence or tolerability issues on the newly started regimen. In a further sensitivity analysis we modified the main inclusion criterion by including patients who started the new regimen with a CD4 count <200 cells/µl (instead of 350) and consequently used this lower cut-off for the definition of time-dependent immuno-virological discordance.

The likelihood ratio test was used to test temporal trends and, more in general, to compare the difference in goodness of fit of nested models (for example models with or without specific interaction terms). STATA v12.1 was used for all analyses. All p-values were two sided and a value of <0.05 was considered significant unless it was a subgroup analysis for which we required the p-value to be smaller than 0.05/k where k was the total number of identified groups (Bonferroni correction).

## Results

**Figure 1:**
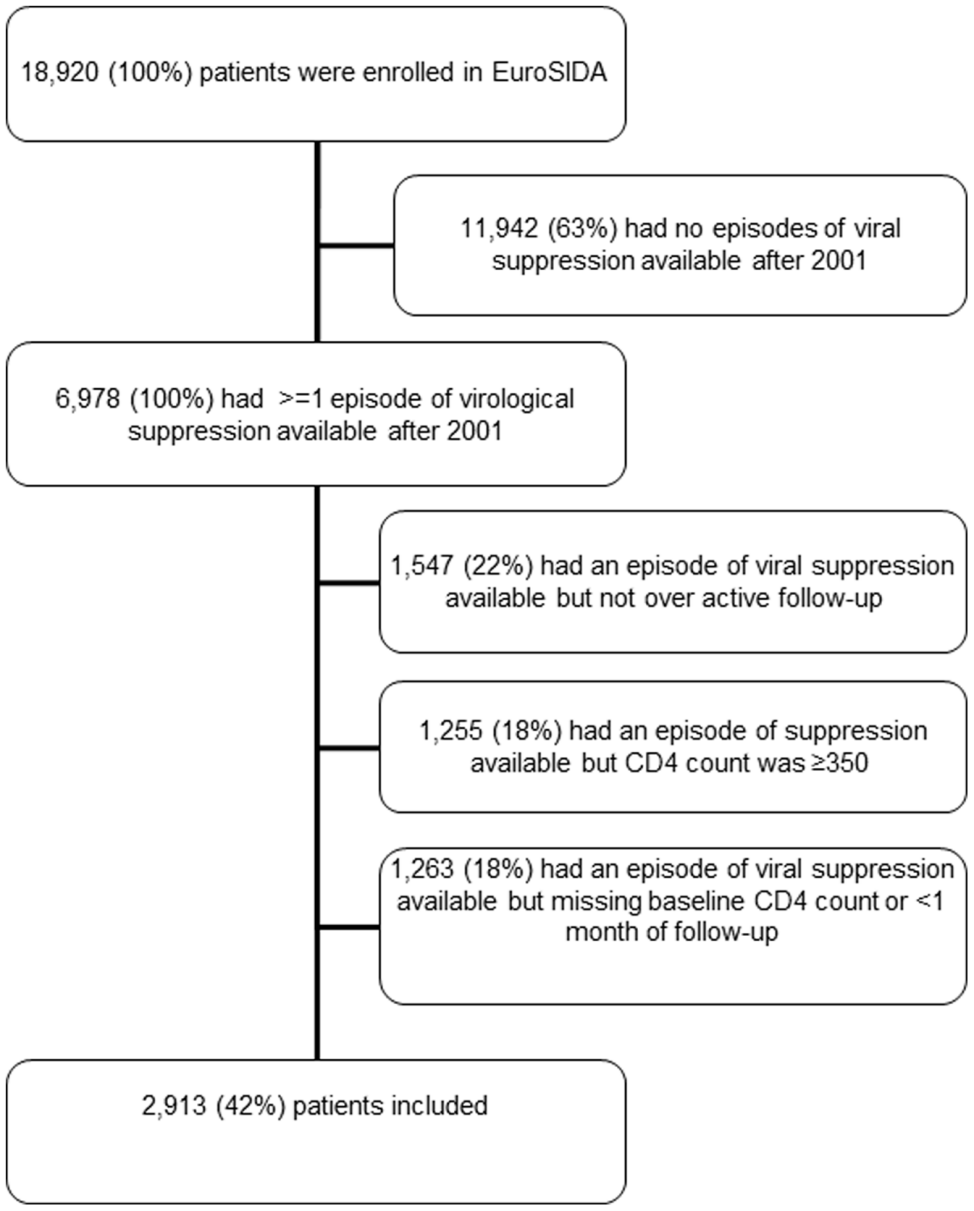
Flow chart showing selection of patients enrolled in EuroSIDA for the analysis.

A total of 2,913 patients satisfied the inclusion criteria for this analysis (Figure 1). Median age was 42 (range 20–82) years. Seventy two percent of patients were male and the most common transmission risk was men having sex with men (38%). At time of initiation of the new regimen the majority of patients (55%) had a CD4 count of 200–350 cells/µl and 25% had never previously received antiretroviral treatment. One third of patients (33%) had experienced clinical AIDS prior to starting the new regimen. These and other characteristics of patients at initiation of the new regimen (baseline) are shown in Table 1.

**Table 1:** Patient characteristics.

	CD4 count 0–99 cells/µl		CD4 count 100–199 cells/µl		CD4 count 200–350 cells/µl	
	No.	%	No.	%	No.	%
*Gender:*						
Male	415	14.2	562	19.3	1,128	38.7
Female	119	4.1	218	7.5	471	16.2
*Ethnicity:*						
Caucasian	462	15.9	661	22.7	1,382	47.4
Asian	13	0.4	10	0.3	33	1.1
Afro-american	42	1.4	57	2	104	3.6
Unknown	17	0.6	52	1.8	80	2.7
*Transmission risk:*						
MSM	168	5.8	285	9.8	663	22.8
IDU	127	4.4	204	7	349	12
Heterosexual	197	6.8	231	7.9	474	16.3
Unknown	42	1.4	60	2.1	113	3.9
*Antiretroviral therapy naïve* *:						
No	378	13	563	19.3	1,233.0	42.3
Yes	156	5.4	217	7.4	366	12.6
*Currently smoking* †:						
No	190	6.5	252	8.7	577	19.8
Yes	197	6.8	274	9.4	586	20.1
Unknown	147	5	254	8.7	436	15
*Hepatitis B coinfection* †:						
No	434	14.9	649	22.3	1,334.0	45.8
Yes	38	1.3	57	2	123	4.2
Unknown	62	2.1	74	2.5	142	4.9
*Hepatitis C coinfection* †:						
No	326	11.2	462	15.9	1,028.0	35.3
Yes	129	4.4	212	7.3	377	12.9
Unknown	79	2.7	106	3.6	194	6.7
*Diabetes mellitus* ‡:						
No	470	16.1	690	23.7	1,433.0	49.2
Yes	17	0.6	28	1	54	1.9
Unknown	47	1.6	62	2.1	112	3.8
*Arterial hypertension* §:						
No	319	11	493	16.9	978	33.6
Yes	121	4.2	152	5.2	388	13.3
Unknown	94	3.2	135	4.6	233	8
*Anaemia* ¶:						
No	262	9	455	15.6	1,006.0	34.5
Yes	210	7.2	257	8.8	448	15.4
Unknown	62	2.1	68	2.3	145	5
						
*Impaired kidney function (GFR<60)* **:						
No	518	17.8	761	26.1	1,573.0	54
Yes	16	0.5	19	0.7	26	0.9
*Previous AIDS event* †:						
No	231	7.9	521	17.9	1,189.0	40.8
Yes	303	10.4	259	8.9	410	14.1
*Previous non-AIDS event* †:						
No	510	17.5	749	25.7	1,527.0	52.4
Yes	24	0.8	31	1.1	72	2.5
*On combination ART* *:						
No	126	4.3	183	6.3	392	13.5
Yes	408	14	597	20.5	1,207.0	41.4
	**Median**	**Range**	**Median**	**Range**	**Median**	**Range**
Age (years)*	43	23–76	43	20–80	42	20–82
CD4 count at start of new regimen (cells/µl)	47.5	1–99	153	100–199	270	200–350
CD4 count at day of suppression (cells/µl)	206.5	6–1,056	270	35–1,064	380	30–1,492
CD4 nadir (cells/µl)	34	1–99	120.5	1–199	202	1–350
Viral load (log cop/ml)†	5.1	2.7–7.8	4.7	2.7–6.9	4.4	2.7–8.1
Year of first viral suppression <50 cop/ml†	2006	2001–2011	2006	2001–2011	2006	2001–2011
Number of previous antiretroviral drugs*	3	0–17	4	0–16	4	0–18

* at start of new regimen.

† at day of first viral suppression (baseline).

‡ diagnosis of insulin dependent diabetes at baseline or having previously started any antidiabetic treatment.

§ systolic/diastolic blood pressure> = 140/> = 90 mmHg mmHg at baseline or previous initiation of any antihypertensive treatment.

¶ haemoglobin <14 mg/dl in males and <12 mg/dl in females at baseline.

** estimated Cockroft-Gault glomerular filtration rate <60 mg/dL/1.73 m^2^ at baseline.

### Fatal and Non-fatal Non-AIDS

**Figure 2:**
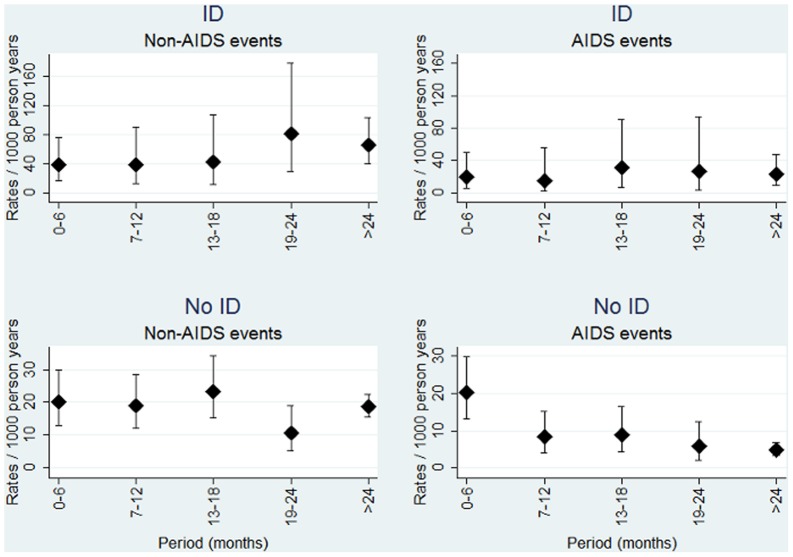
Rates (and 95% confidence intervals) of fatal and non-fatal non-AIDS and AIDS in patients with and without immuno-virological discordance (ID).

For the primary endpoint, fatal and non-fatal non-AIDS, 11,491 person years of follow-up were available and 241 events including 157 non-AIDS disease events and 84 non-AIDS deaths occurred. Malignancies classified as not AIDS related were the most common disease events (n = 72, 46%) followed by cerebrovascular diseases (n = 46, 39%). Kaplan Meier estimate of median survival time in patients who died was 2.1 (95% CI 1.6–2.8) years. Malignancies (n = 23, 27%) were also the most common causes of death. 42 (17%) fatal or non-fatal non-AIDS events occurred in ID patients (rate 53/1,000PYFU [95% CI 38–72]) vs. 199 (83%) in patients without ID (rate 19/1,000PYFU [95% CI 16–21]) p<0.001 (Figure 2).

In the adjusted Poisson regression analysis including demographic and clinical parameters (Table 2; model 1) the risk of fatal and non-fatal non-AIDS was almost two-fold higher in patients currently classified as ID compared to those classified as non-ID (aRR 1.96, 95% CI 1.37–2.81, p<0.001) Point estimates were slightly lower in a model adjusted for demographic factors as well as baseline CD4 count (Table2; model 2). After adjusting for current CD4, only a tendency for an association between ID and the risk of non-AIDS remained (aRR 1.43, 95% CI 0.94–2.17,p = 0.095).

**Table 2:** Multivariable Models with different endpoints and adjusted for demographic factors alone (model 1), demographic factors and baseline CD4 count (model 2), and demographic factors and current CD4 count (model 3).

Fatal and non-fatal non-AIDS																					
						Univariable Analysis				Multivariable Model 1§				Multivariable Model 2§				Multivariable Model 3§			
	Events	FU	Rates	95% CI		IRR	95% CI		p	IRR	95% CI		p	IRR	95% CI		p	IRR	95% CI		p
non- ID*	199	10,699	18.6	16.1	21.4	1	1	1	–	1	1	1	–	1	1	1	.	1	1	1	–
ID*	42	792	53.1	38.2	71.7	2.85	2.06	3.96	<0.001	1.96	1.37	2.81	<0.001	1.94	1.32	2.85	0.001	1.43	0.94	2.17	0.095
Baseline CD4 count†														0.90	0.78	1.04	0.151				
Current CD4 count‡																		0.78	0.65	0.92	0.004
**Fatal and non-fatal AIDS**																					
						**Univariable Analysis**				**Multivariable Model 1** §				**Multivariable Model 2** §				**Multivariable Model 3** §			
	**Events**	**FU**	**Rates**	**95% CI**		**IRR**	**95% CI**		**p**	**IRR**	**95% CI**		**p**	**IRR**	**95% CI**		**p**	**IRR**	**95% CI**		**p**
non-ID*	81	10,903	7.4	5.9	9.2	1	1	1	–	1	1	1	–	1	1	1	–	1	1	1	–
ID*	18	819	22	13	34.7	2.96	1.78	4.92	<0.001	1.86	1.07	3.23	0.028	2.27	1.26	4.07	0.006	0.76	0.41	1.38	0.361
Baseline CD4 count†														0.68	0.53	0.86	0.002				
Current CD4 count‡																		0.50	0.40	0.62	<0.001

* Immuno-virological Discordance.

† per 100 cells/µl higher.

‡ per log2 cells/µl higher.

§ multivariable Poisson regression also adjusted for age, sex, transmission risk, race, region of cohort, calendar year of baseline, treatment naive/experienced, current or previous smoking, hepatitis B/C coinfection, whether or not on triple ART, number of treatment changes, diagnosis of anaemia, hypertension, impaired renal function, viral load at start of new regimen, time to viral suppression, previous AIDS or non-AIDS event.

Compared to the first 6 months of viral suppression (reference group), the adjusted relative rate of experiencing fatal or non-fatal non-AIDS for patients classified as ID was 1.06 (95% CI 0.32–3.50) in month 7–12, 0.81 (95% CI 0.23–2.81) in month 13–18, 1.67 (95% CI 0.51–5.41) in month 19–24 and 0.91 (95% CI 0.23–3.60) thereafter, test for trend p = 1.00). Similarly, there was no tendency for a decline in risk in periods in which patients were classified as non-ID (month 7–12: aRR 0.97, 95% CI 0.55–1.72, month 13–18 aRR 1.25, 95% CI 0.71–2.18, month 19–24 aRR 0.59, 95% CI 0.28–1.23, and >24 months aRR 1.14, 95% CI 0.63–2.07, p-value for test of trend = 0.24).

Factors besides ID and current CD4 count significantly associated with the risk of developing the composite endpoint in the main analysis included older age, a diagnosis of anaemia prior to baseline, previous non-AIDS, and current use of antiretroviral treatment consisting of less than 3 drugs (Table 3).

**Table 3:** Other factors associated with fatal and non-fatal non-AIDS (only factors with p<0.1 are shown).

Multivariable Analysis*				
	IRR	95% CI		p
Intravenous drug use (vs. MSM)	1.53	0.98	2.39	0.06
Currently on triple ART	0.69	0.5	0.96	0.03
Diagnosis of anaemia†	2.11	1.62	2.75	<0.001
Age (per 10 years older)	1.7	1.47	1.97	<0.001
Previous non-AIDS event(vs. no event)	1.65	1.02	2.65	0.04
Current CD4 count(per log2 cells/µl higher)	0.78	0.65	0.92	0.004

* multivariable Poisson regression also adjusted for immune-virological discordance status, region of cohort, treatment naive/experienced, current or previous smoking, hepatitis B/C coinfection, transmission risk, race, sex, calendar year of baseline, number of treatment changes, diagnosis of hypertension, diagnosis of diabetes, diagnosis of impaired renal function, viral load at start of new regimen, time to viral suppression, and previous AIDS. CD4 count at baseline is not included in the model.

† haemoglobin <14 mg/dl in males and <12 mg/dl in females.

### Sensitivity Analyses for the Non-AIDS Endpoint

The association between ID and the risk of non-AIDS events appeared to be higher in patients who were therapy-naïve (aRR = 3.06, 95% CI 0.69–13.55) vs. those who were treatment experienced (aRR = 1.30, 95% CI 0.84–2.02) at baseline, although there was no evidence of interaction (p-value = 0.583).

After restricting our analysis to patients who achieved viral suppression within one year after initiation of cART (n = 1,489, 51% of total follow-up for 6,333 person years and 162 events), the association between ID and the risk of developing non-AIDS events (aRR 1.63, 95% CI 0.95–2.78, p = 0.073) was consistent with that found in the main analysis. In a sub-analysis of ART naïve patients who achieved complete virological suppression within one year (21 events only), ID was marginally significantly associated with non-AIDS even after controlling for current CD4 (aRR for ID 4.54, 95% CI 0.97–21.2, p = 0.054).

Results were similar in a sensitivity analysis defining virological failure as one viral load >50 regardless of confirmation (data not shown).

In the analysis using the alternative cut-off of 200 CD4 cells/µl (n = 1,349, 170 events, 5247 PYFU), results were also similar: aRR 2.39, 95% CI 1.37–4.18, p = 0.002) in the model adjusted only for demographic and clinical factors and aRR 1.58, 95% CI 0.81–3.12, p = 0.183) after further controlling for current CD4 count.

### Fatal and Non-fatal AIDS

In the separate analysis with AIDS as an endpoint, 11,723 person years of follow-up were available. A total of 89 AIDS events including 10 AIDS-related deaths occurred. Oesophageal candidiasis was a frequently observed opportunistic infection (n = 15, 17%) followed by pulmonary and extrapulmonary tuberculosis (14 patients, 16%). Kaplan Meier estimate of median survival time in patients who died was 1.3 (95% CI 0.3–2.0) years. AIDS malignancies were the most common cause (n = 8, 80%).

ID was significantly associated with the risk of developing AIDS-related events when adjusted for demographic and clinical factors only (aRR 1.86, 95% CI 1.07–3.23, p = 0.03). However, after adjusting for current CD4 count, ID was no longer independently associated (aRR 0.76, 95% CI 0.41–1.38, p = 0.36). Factors significantly associated with the risk of developing AIDS included current CD4 (aRR 0.50 per doubling of the count, 95% CI 0.40–0.62, p<0.001), diagnosis of anaemia at baseline (aRR 2.21, 95% CI 1.46–3.35, p<0.001), and a previous AIDS condition (different from that observed in follow-up, aRR 2.07, 95% CI 1.33–3.20, p = 0.001).

## Discussion

In our analysis, we found that people who failed to achieve a CD4 count above the level present at the time of starting a new regimen, despite having achieved and maintained viral suppression <50 copies/mL on ART, tended to have an increased risk of developing non-AIDS morbidity and mortality. Because of the wide confidence interval around our estimate, our results may be considered inconclusive. However, a two-fold increased risk in people with ID cannot be excluded. Our results should guide further investigations using larger study populations to test whether the lack of statistical significance was due to limited power of our analysis. Of note, the association was independent of demographic characteristics and baseline CD4 count but seemed to be mainly explained by a low current CD4 count. There was a small residual independent effect but only in people who started the new regimen when they were ART-naïve and who achieve virological suppression within one year. In additions, there was no evidence for a decreasing risk of non-AIDS events with longer time with a viral load < = 50 copies/mL in people with or without ID.

Our data suggest that in order to reduce the risk of non-AIDS events in people starting therapy with a CD4<350 cells/µl, continuous monitoring for difference between current CD4 count and the level registered at the time of initiation of a new regimen may be considered starting as early as the first 6 months following viral suppression. The cut-off of 350 CD4 cells/µl as baseline was chosen as this represents the threshold below which cART is generally recommended. However, also when the alternative cut-off of 200 CD4 cells/µl was used, our results were similar although the analyses had reduced statistical power.

In a previous analysis, ID was associated with a higher risk of AIDS and death [10]. However, this analysis was unadjusted for current CD4 count and only included patients initiating their first cART from ART-naïve at <200 CD4 cells/µl in a cohort in Germany. Because of the different inclusion criteria as well different definitions of ID patients, results cannot be directly compared.

Previous analyses of EuroSIDA data demonstrated that current CD4 count is a significant predictor of the risk of non-AIDS events although the association was less strong than that found for AIDS events [11], [17]. More specifically, it was previously shown, that those with a current CD4 count of <200 cells/µl and with suppressed viraemia on cART have lower rates of AIDS and, to a lesser extent, non-AIDS events compared to patients with ongoing viral replication [18]. The potential independent role of ID had not been investigated in this analysis.

In the present study we included time-updated current CD4 count and immuno-virological discordance status, because we hypothesized that the risk associated with the incapacity to reconstitute CD4 cells over time may not be fully captured by current CD4 count without taking into account the status of immunosuppression observed prior to starting the suppressive regimen. A similar approach was also used in a recent study by Lapadula et al who, however, used a different definition of non-AIDS events but a similar definition of ID patients and found a strong association between lack of CD4 count recovery on suppressive ART and risk of developing the composite endpoint of AIDS and non-AIDS events [19].

The finding for non-AIDS events is intriguing as it suggests that patients’ current status of immunodeficiency is not able to fully explain why some people remain at higher risk of clinical complications. However, this needs to be confirmed in further analyses as measurement variability in CD4 tests and differences in frequencies of measurement may be alternative explanations.

Indeed, it is possible that ID is a marker of one of more unmeasured factors that put these people at increased risk of an adverse clinical outcome. For example, immuno-virological discordance may be the effect of incomplete bone marrow or thymus output as well as excessive destruction or disturbed immunoregulation. [20] In addition, lymph node fibrosis could contribute to persistent low CD4 counts. [21] Furthermore, higher mortality rates were recently observed in parents of immunological non-responders suggesting that genetic factors may be involved as well [22]. In patients with low CD4 counts but suppressed viraemia higher rates of cellular immune activation markers including CD38 and HLA-DR together with higher cell-associated HIV-RNA have been observed and this has been linked particularly to non-AIDS morbidity and mortality [23]. The higher immune activation may set the stage for non-AIDS events including cardiovascular disease [24]. Furthermore, non-AIDS malignancies have been strongly linked with HIV induced immunodeficiency [25], [26]. In our analysis, we failed to show an effect of ID which was independent of current CD4 count. This can be due to lack of power or characteristics of the study population included. Indeed, when restricting to fully adherent persons who started a new regimen from ART-naïve we obtained stronger evidence for an independent effect. Of note, in contrast, there was no significant association between ID status and the risk of AIDS events.

Our study has some limitations: first, the non-AIDS conditions considered here as endpoint-defining events represent a subset of non-AIDS events causing HIV morbidity. However, this selection has been previously used [11]. Second, based on our restrictive inclusion criteria, the numbers of people with an event is quite low and we were not able to investigate the risk associated with specific groups of conditions separately (for example we could hypothesize that the risk associated with being ID varies by specific malignancy). In addition, it was not possible to obtain robust estimates of the event rates after 24 months from baseline in the ID group due to the low number of patients remaining at risk after this time point.

In conclusion, patients with immuno-virological discordance, in the context of suppressed viral load appeared to be at higher risk of fatal and non-fatal non-AIDS events although most of this increased risk seems to be explained by a low current CD4 count. An independent effect was found only in the subset of ART-naïve patients who experienced a rapid virological response. Another important finding was that this increased risk, if it exists, does not seem to decrease with longer duration of viral suppression, suggesting that such patients should remain under close follow-up for change in CD4 count from pre-therapy level, also after extended periods of being on cART. If this observation is confirmed in other studies it has profound implications as it suggests that cART alone may not be able to substantially remove the risk of non-AIDS in patients identified as ID. To date, strategies to directly influence immune reconstitution by adding interleukin-2 or by modifying cART regimens have failed to show benefit over viral suppression alone [27], [28]; therefore new strategies perhaps aiming at other mechanisms to boost functional CD4 cells or decreasing the levels of immune-activation (e.g. interleukin-7, probiotics) need to be tested in people who show incomplete immune reconstitution despite sustained viral suppression.
